# Intraductal laser ablation during ductoscopy in patients with pathological nipple discharge

**DOI:** 10.1007/s10549-024-07568-4

**Published:** 2025-02-04

**Authors:** Seher Makineli, Menno R. Vriens, Paul J. van Diest, Arjen J. Witkamp

**Affiliations:** 1 Department of Surgical Oncology, University Medical Center, PO Box 85500, 3508 GA Utrecht, The Netherlands; 2 Department of Pathology, University Medical Center, PO Box 85500, 3508 GA Utrecht, The Netherlands

**Keywords:** Breast neoplasms, Nipple discharge, Ductoscopy, Laser

## Abstract

**Background:**

Ductoscopy is a minimally invasive micro-endoscopic approach for direct visualization and removal of intraductal lesions of the breast. A challenge of ductoscopy is an adequate treatment of intraductal lesions by complete removal to prevent exploratory duct excision surgery. This study aimed to determine the in vivo feasibility of intraductal laser ablation during ductoscopy to remove intraductal lesions in patients suffering from pathological nipple discharge (PND).

**Methods:**

A prospective, single-center diagnostic feasibility trial was conducted between October 2022 and November 2023, enrolling adult women with unilateral PND and no radiological suspicion of malignancy. Intraductal laser ablation was performed after incomplete intraductal biopsy using a Thulium laser.

**Results:**

Duct cannulation and subsequent ductoscopic exploration were successful in 21 patients revealing an intraductal lesion in 13 patients (61.9%). From these 13 patients, 9 patients (69.2%) underwent intraductal laser ablation due to a residual lesion after biopsy. Pathology of the removed intraductal lesions showed a papilloma in eight (88.9%) patients and a papilloma/DCIS combination in one patient (11.1%). Post-procedure, PND stopped in 77.8% of the patients (7/9). Two patients had recurrent PND complaints caused by a residual lesion.

**Conclusion:**

Intraductal laser ablation during ductoscopy in patients with papillary lesions seems to be feasible and safe. The Thulium laser enables ablation of residual lesions and is therefore suitable for an immediate second intervention after ductoscopic removal of intraductal lesions. Further refinement and validation in a follow-up clinical trial are necessary to further assess its therapeutic efficacy.

## Introduction

Pathological nipple discharge (PND) is a common breast-related condition characterized by unilateral, spontaneous, and bloody or serous discharge arising from a single duct orifice of the nipple [1]. PND is often viewed as a breast cancer sign, but the most common causes of PND by far are benign (ductectasias and intraductal papillomas) [2–4]. Surgical duct excision is traditionally required to rule out malignancy in patients with PND without radiological and clinical abnormalities [5–7]. However, the malignancy rate after duct excision surgery is only 8.1%, meaning that the majority of these surgical procedures (microdochectomy or major duct excision) are performed for benign causes. This can lead to surgery-related complications (1.4%) such as hematomas, surgical site infections, and seromas [8]. Other adverse effects of duct excision surgery include higher costs and the need for more medical personnel [9]. In times of staff shortages and rising healthcare expenses, gains can be achieved with a better selection of patients that actually will benefit from duct excision surgery. Ductoscopy is a minimally invasive micro-endoscopic approach for direct visualization and removal of intraductal lesions of the breast [10]. After mammography and ultrasound, ductoscopy can be performed in the diagnostic work-up for PND patients without radiological abnormalities [11, 12]. A randomized controlled trial found that ductoscopy is as accurate as conus excision in identifying the causative lesion of PND [13]. Additionally, PND patients with non-suspicious conventional imaging and negative ductoscopy have a low malignancy rate, making subsequent microdochectomy unnecessary in 2 out of 3 patients [14, 15]. However, some patients still suffer from PND after ductoscopy, and in most cases, these patients eventually undergo a surgical procedure or a second ductoscopy due to recurrent or persistent PND [16–19].

Current endoscopic interventional methods for PND remain suboptimal. Therefore, there is a need for more effective interventional possibilities of ductoscopy to remove intraductal lesions completely. One promising new intervention is adding laser ablation to the ductoscopy procedure to remove intraductal lesions completely. Laser ablation techniques have been widely used in various medical fields and have been proven to be safe and effective in evaporating lesions [20–23]. Consequently, our research team previously conducted an ex vivo feasibility study of endoscopic intraductal laser ablation of and found that laser ductoscopy is technically feasible and can serve as an adjuvant tool for minimally invasive treatment of (residual) intraductal papillomas in PND patients [24].

As a result, we have conducted an in vivo feasibility study with intraductal laser ablation during ductoscopy in PND patients. The main goal of this study was to determine the in vivo feasibility of intraductal laser ablation in patients with intraductal lesions. To the best of our knowledge, this is the first study to perform intraductal laser ablation during ductoscopy in patients.

## Methods

This study was approved by the Medical Research Ethics Committee of the University Medical Center Utrecht in The Netherlands (METC protocol number 21–688/H-D). All participants provided written informed consent. The study protocol was published in September 2022 [25].

### Study design and population

This phase II, prospective, single-center, diagnostic feasibility trial was conducted at the University Medical Center (UMC) Utrecht in The Netherlands between October 2022 and November 2023. The study population included adult women (≥ 18 years) with unilateral PND and no radiological suspicion of malignancy, who underwent a ductoscopy procedure at UMC Utrecht. Laser ablation was performed when there was an intraductal lesion visible which was incompletely removed using the basket or biopsy tools while tissue for pathology was obtained. PND was defined as unilateral, bloody or serous nipple discharge during a non-lactational period, persisting for at least three months. The exclusion criteria were: pregnancy, previous breast surgery at the affected breast that would make ductoscopy technically impossible, radiotherapy of the breast or thorax, nipple retraction and the impossibility of obtaining tissue sampling from the lesion.

### Data collection

Standard clinical variables were collected, including age at presentation, characteristics of the nipple discharge (laterality and spontaneous versus expressed), physical exam findings (palpable breast mass and productive ducts), and follow-up period. In addition, details of diagnostic methods, imaging studies, and histopathological findings were recorded for each case.

### Work-up

Before ductoscopy, all patients underwent a standard diagnostic evaluation, including a complete medical history and physical examination and imaging (mammography, ultrasonography, and/or MRI) and core needle biopsy when indicated to rule out malignancy.

### Ductoscopy procedure

Ductoscopy was performed in the daily routine at the outpatient clinic. Lidocaine 1% was used for local anesthesia of the nipple. A salivary duct probe (size 0000 to 1; Karl Storz, Tuttlingen, Germany) and an obturator (Polydiagnost, Pfaffenhofen, Germany) were used to widen the lactiferous duct of the nipple to a diameter of 1.2 mm. The SoLex nipple expander® (Polydiagnost), was then inserted through the port into the affected duct. The 6000‐pixel 0.55‐mm optic (LaDuScope T‐flex; Polydiagnost) and the Polyshaft® (1.15 mm outer diameter, PD‐DS‐1015; Polydiagnost) were used for ductoscopy. The Polyshaft® system has three channels: one for the endoscope, one for saline irrigation or additional intraductal anesthetic infusion, and one for the biopsy tool and laser fiber. The surgeon explored the major ducts until they became too narrow to pass. Intraductal biopsies were performed when lesions were identified. The final step of the procedure was intraductal laser ablation, which was performed when the lesion was incompletely removed using the biopsy tools. A MED-fiber (Tobrix, Waalre, The Netherlands) with a core diameter of 200 µm and an outer diameter of 375 µm was introduced through the working channel. Laser energy was delivered using 2013 nm thulium laser generator (Revolix Junior; LISA Laser Products, Katlenburg, Germany) at power settings of 1–4 W with single pulses of 100–1000 ms. Laser ablation was applied until no visible vital tissue of the lesion to be treated remained.

All patients were followed at least after two weeks and three months after ductoscopy to evaluate the effect of treatment on symptoms.

### Statistical analysis

Prevalence and means with standard deviation (SD) were calculated with SPSS v29.0 to describe the study population.

## Results

### Baseline characteristics

**Fig. 1:**
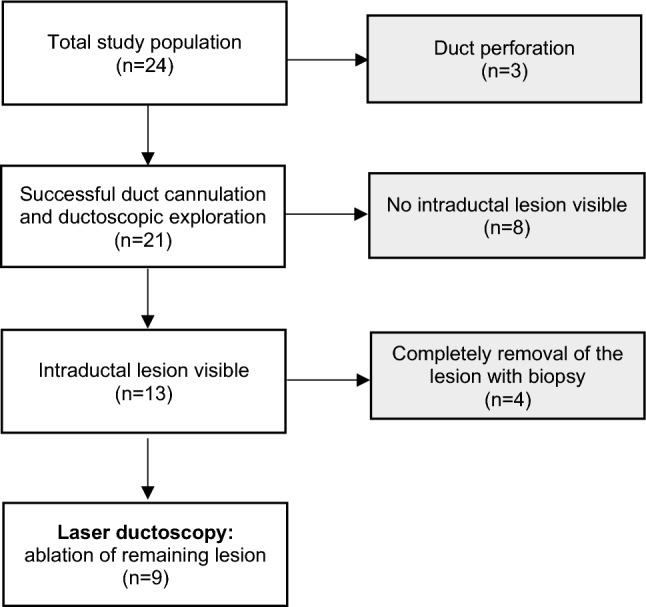
Flowchart of the study population

From October 2022 to November 2023, a total of 24 patients met the inclusion criteria. Duct cannulation and subsequent ductoscopic exploration were successful in 21 patients (87.5%), revealing an intraductal lesion in 13 patients (61.9%). Biopsy samples were successfully obtained from all patients with intraductal lesions using a biopsy tool or basket. Finally, 9 patients (42.9%) underwent intraductal laser ablation due to a remaining lesion after biopsy (Fig. 1).

The mean age of the patient population at the time of ductoscopy procedure was 53.4 ± 10.7 years. Clinical data of the patients are presented in Table 1. Unilateral discharge was noted in all cases, and spontaneous discharge was observed in 8/9 cases. All patients presented with single duct PND with bloody, yellow/brown or a clear color.

**Table 1:** Clinical data of 9 patients with pathological nipple discharge undergoing laser ablation

Clinical findings	No. N = 9
Age, mean ± SD, years	53.4 ± 10.7
Affected breast, N (%)	
Left	5 (55.6)
Right	4 (44.4)
Palpable abnormality, N (%)	3 (33.3)
Color nipple discharge	
Clear Yellow/brown Bloody	1 (11.1) 2 (22.2) 6 (66.7)
Ultrasound findings, N (%)	
BI-RADS 1	1 (11.1)
BI-RADS 2	4 (44.4)
BI-RADS 3	3 (33.3)
BI-RADS 4a	1 (11.1)
Mammographic findings, N (%)	
BI-RADS 2	3 (33.3)
BI-RADS 3	5 (55.6)
Not performed	1 (11.1)
MRI findings, N (%)	
BI-RADS 2	3 (33.3)
BI-RADS 3	2 (22.2)
Not performed	4 (44.4)
Pathology before ductoscopy, N (%)	
No abnormalities	2 (22.2)
Papilloma	1 (11.1)
Ductectesia	3 (33.3)
Not performed	3 (33.3)
Cytology PND, N (%)	
No abnormalities	4 (44.4)
Papilloma	1 (11.1)
Cystic cells	1 (11.1)
Not performed	3 (33.3)

*SD* standard deviation; *MRI* magnetic resonance imaging; *PND* pathological nipple discharge

Ultrasound was conducted as part of the standard evaluation in all patients. The results revealed normal findings or lesion(s) with a low suspicion of malignancy. Mammography was performed in 8/9 cases. Furthermore, MRI was performed in 5 cases, with two cases indicating normal findings, two cases with duct ectasia and one patient suspected of an intraductal papilloma. 6 out of 9 patients underwent core needle biopsy prior to ductoscopy.

### Power settings

Table 2 presents an overview of the outcomes derived from laser ductoscopy. Laser ablation was carried out using single pulses of 100–1000 ms. Throughout the process of laser ablation, an endoscopic perspective was consistently maintained, utilizing power levels ranging from 1 to 4 Watts (W). Notably, at 1W, a mild impact was observable on the intraductal papilloma. Upon escalation to 3W, shrinkage of the intraductal papilloma was achieved. Increasing power to 4W resulted in a more pronounced reduction, although this power was not needed for the majority of procedures. The total energy used by removal of the intraductal papilloma ranged from 31 to 226 Joules (J). The duration of laser ablation ranged from 0.21 to 1.12 min.

**Table 2:** Overview of 9 patients with pathological nipple discharge that underwent intraductal laser ablation

Patient	Age	Nipple discharge	Radiology (BI-RADS)	Ductoscopic findings	Intraductal extraction of lesion	Laser setting	Pathology	Follow-up after 3 months
1	53	bloody	US + MG + MRI: 2	Polypoid lesion	basket	2.0 W / 133 J 1.09 min	Intraductal papilloma	Remaining lesion: recurrence of PND. Duct excision surgery
2	37	bloody	US + MG: 3	Polypoid lesion	basket	2.0 W / 31 J 0.21 min	Intraductal papilloma with foci ADH/DCIS	Successful treatment: Follow-up with mammogram
3	50	clear	US + MG: 3	Polypoid lesion	biopsy tool	3.0 W / 120 J 0.50 min	Intraductal papilloma	Successful treatment
4	67	bloody	US + MG + MRI: 3	Polypoid lesion	basket	3.0 W / 80 J 0.32 min	Intraductal papilloma	Successful treatment
5	51	bloody	US + MRI: 4a	Polypoid lesion	basket	4.0 W / 226 J 1.12 min	Intraductal papilloma	Successful treatment
6	67	Yellow / brown	US + MMG + MRI: 3	Polypoid lesion	biopsy tools	3.0 W / 80 J 0.31 min	Intraductal papilloma	Successful treatment
7	45	bloody	US + MG: 3	Polypoid lesion	basket	3.0 W / 101 J 0.45 min	Intraductal papilloma	Remaining lesion: recurrence of PND. Re-laser
8	65	bloody	US + MG + MRI: 2	Polypoid lesion	basket	3.0 W / 53 J 0.22 min	Intraductal papilloma	Successful treatment
9	45	yellow	US + MG: 3	Polypoid lesion	basket	4.0 W / 205 J 1.07 min	Intraductal papilloma	Successful treatment

*BI-RADS* breast imaging reporting and data system; *US* ultrasound; *MG* mammography; *MRI* magnetic resonance imaging; *W* watt, *J* joule; *ADH* atypical ductal hyperplasia; *DCIS* ductal carcinoma in situ

### Histopathological findings

**Fig. 2:**
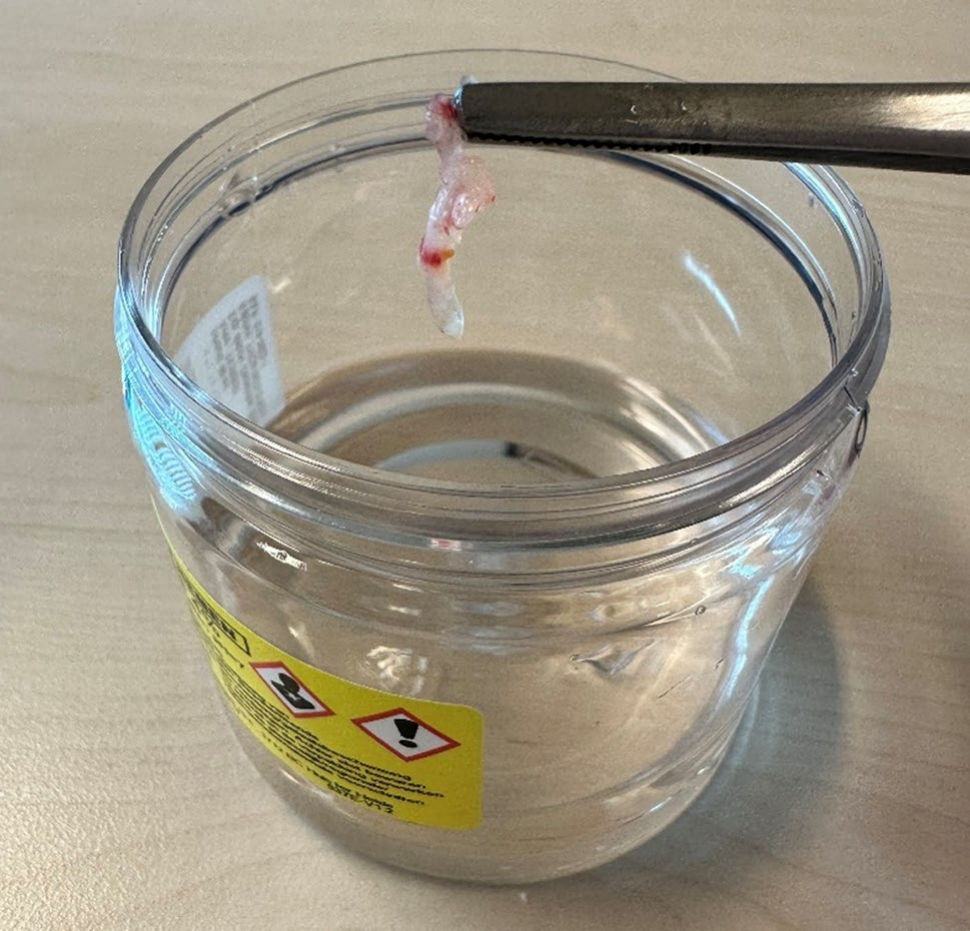
The removed intraductal lesion in patient 2 with a focus of ADH/DCIS. The remaining lesion in the milk duct was ablated with the Thulium laser

Pathology of the biopted tissues showed an intraductal papilloma in 8 patients. One patient (11.1%) experienced PND due to an intraductal papilloma with a focus of ADH/DCIS (Fig. 2). Post-procedure, there was no visibly remaining lesion due to complete laser ablation. In this case, follow-up with mammography will be carried out.

### Follow-up

Follow-up data were available for all included patients. During ductoscopy, laser ablation was performed until no visible vital tissue of the target lesion remained. After successful laser ductoscopy, PND stopped in 7 out of 9 (77.9%) patients. However, two patients experienced recurrent PND complaints, due to a remaining lesion. One of these patients underwent a second ductoscopy with laser ablation, which revealed a residual lesion, while the other patient opted for duct excision surgery. Duct excision surgery (microdochectomy / major duct excision) could thereby be avoided in 8/9 (88.9%) patients.

One patient experienced post-procedural pain in the nipple for 1 week. Post-procedure, an MRI was performed without any abnormalities. The remaining patients did not experience any post-procedural pain or other side effects. No other complications were reported.

## Discussion

The aim of this study was to assess the feasibility of laser treatment for intraductal papillomas causing PND. This interventional study demonstrated that intraductal laser ablation during ductoscopy was technically feasible in patients with intraductal lesions. The Thulium laser was capable of evaporating intraductal papillary lesions in cases with remaining lesions after biopsy resulting in discontinuation of PND complaints in 77.8% after treatment in the follow-up period of three months. There were no complications, and only 1 patient complained of post-procedural nipple pain, which can also generally be seen after ductoscopy so this cannot with certainty be attributed to the laser ablation. Laser ductoscopy thereby has potential to safely improve the therapeutic intervention capability of ductoscopy in patients with benign intraductal lesions and successfully prevent unnecessary exploratory surgery. However, further refinement and validation in follow-up clinical trials are necessary.

Ductoscopy enables the detection of malignancies with a specificity of 92% and a sensitivity of 58% [26]. Although current intraductal biopsy tools can remove lesions during ductoscopy, their removal often remains incomplete. [16, 27]. According to a prior study conducted by our research team, removal of the lesion was possible in only 36.8% of the study population [14]. In these cases, in which tissue sampling from the lesion can be obtained, laser ablation serves as a promising addition to the therapeutic capabilities of ductoscopy while retaining histological confirmation. In the present study, laser ductoscopy made it possible to remove intraductal lesions in 77.8% of patients with remaining intraductal lesions after basket removal. After undergoing regular ductoscopy, patients can still suffer from PND and therefore undergo a surgical procedure or a second ductoscopy [16–18]. According to a cohort study, persistent or recurrent PND after a first ductoscopy procedure was primarily caused by a remaining intraductal papilloma in the majority of patients (95%) [19]. In such cases, if laser ductoscopy was performed during the primary ductoscopy procedure, complete removal of the intraductal lesion may have been possible in a greater number of patients, thereby potentially avoiding a second (surgical) intervention.

Laser ductoscopy can improve the patient selection process for surgical procedures in the workup of PND without clinical or radiological abnormalities, because successful (laser) sablation prevents the necessity for further invasive procedures [14]. However the presence of an intraductal mass is a possible predictor for malignancy, so definitive histological diagnosis is mandatory before performing laser ablation [15]. Consequently, laser ductoscopy can lead to a reduction of the need for additional surgery and fewer surgery-related complications such as hematomas, surgical site infections, and seromas [8].

However, the role of laser ductoscopy in cases of PND caused by intraductal DCIS or invasive cancer is uncertain. In this study, one patient experienced PND due to an intraductal papilloma with a focus of ADH/DCIS grade 1. Following an intraductal biopsy during ductoscopy, laser ductoscopy was performed. Post-procedure, the localization of the tumor site for surgical resection by wide local excision was not possible because there was no remaining lesion on imaging due to complete removal with laser ablation. In this case, follow-up with mammography will be carried out. Given that observation for low-grade DCIS is becoming more common and the natural progression of ADH or DCIS within a papilloma is not well known, this may be an acceptable risk when paired with clinical and imaging surveillance. The potential for laser ductoscopy to become a routine intervention for premalignant breast lesions remains speculative. Ongoing clinical trials, such as LORD, LORIS, COMET, and LORETTA, are investigating whether low-risk DCIS is overtreated and if active monitoring is a safe approach [28]. Preliminary results suggest that active surveillance for low-risk DCIS is feasible. This supports the possibility that follow-up after laser ablation of low-grade DCIS within a papilloma may be safe. However, long-term outcomes are awaited.

According to our findings, laser ductoscopy can be safely integrated into the diagnostic and therapeutical approach for pathological nipple discharge to remove intraductal lesions in patients with remaining intraductal lesions after basket removal and subsequent histological biopsy. This procedure can be incorporated into the initial ductoscopy procedure in the presence of a visible residual lesion. Additionally, it can also be performed during a second ductoscopy procedure in patients with recurrence of complaints due to a remaining lesion. Laser ductoscopy can be implemented in medical centers already performing ductoscopy procedures for pathological nipple discharge. The widespread adoption of this technique into the work-up of PND, particularly in centers performing duct excision surgery, holds promise for the future.

To our knowledge, this is the first study to report on the application of intraductal laser ablation within a ductoscopy procedure. However, certain limitations do warrant consideration. Given the design of this study as a feasibility study, it features a relatively small sample group size of included patients. This study clearly showed the feasibility of intraductal laser ablation during ductoscopy using a Thulium laser. Nevertheless, to comprehensively evaluate both diagnostic accuracy and therapeutic efficacy, further refinement and validation in clinical trials are necessary. Additionally, the identification of optimal power settings for achieving adequate removal, as well as an examination of the effects of using different types of lasers (e.g., Holmium vs. Thulium laser) on intraductal papillomas, will have to be studied [29, 30]. Moreover, differentiation between ADH and low-grade DCIS is based on size criterion of 3 mm in papillary lesions. Therefore, due to the small size of the biopted tissue, definitive categorization may not always be possible.

To conclude, laser ablation during ductoscopy is safe and feasible in for evaporating residual intraductal breast lesions. This technique holds the potential to enhance the minimally invasive therapeutic intervention capabilities of ductoscopy procedures for patients suffering from PND without other clinical or radiological abnormalities.

## Data Availability

No datasets were generated or analysed during the current study.
